# Effectiveness of Environmental Design Interventions to Reduce Aggression and Violence in Emergency Departments: A Scoping Review

**DOI:** 10.1177/19375867251351027

**Published:** 2025-07-07

**Authors:** Darren Jacob, Belinda Jacob, Elisabeth Jacob, Alycia Jacob

**Affiliations:** 1 95359 Australian Catholic University, Australia

**Keywords:** emergency department, violence, physical design, effectiveness, aggression

## Abstract

**Aim:**

To investigate evidence for the effectiveness of physical design interventions to reduce patient and bystander violence in emergency departments.

**Background:**

Workplace violence in emergency departments can cause financial, emotional and physical harm for health care staff and organizations. Violence may be impacted by the physical design of the department.

**Method:**

A scoping review was undertaken of CINAHL, Medline, Scopus, and PsycINFO. The search utilized Boolean operators with key words, major search terms and subject headings. Inclusion criteria were physical design, violence and emergency departments. Studies on mental health or pediatric emergency departments, or non-research papers were excluded. AS review was used to sort and filter. Data was extracted into Covidence. Studies were reviewed for physical design elements used to manage aggression and outcomes. Content analysis of extracted data identified four themes.

**Results:**

Ten papers were identified. The majority of studies provided staff perceptions on the effectiveness of physical design on violence. Data revealed four main areas where physical design interventions could impact on violence from patients and bystanders. These were preventing harm from weapons, controlling physical access, observation and awareness and patient comfort.

**Conclusion:**

There is little evidence for the effectiveness of physical design interventions to reduce patient and bystander violence in hospital emergency departments. Despite a lack of empirical evidence, staff perceive that the physical design of the emergency department impacts on their safety. Further research is needed to better understand the effect of physical design on violence and determine which interventions are effective in impacting on aggressive behavior.

## Introduction

Workplace violence (WPV) in healthcare is an increasing problem worldwide (Liu et al., 2019; Hendrickson, 2022; MohammadiGorji et al., 2021). Safe Work Australia (2024, p. 1) define WPV as “any incident where a person is abused, threatened or assaulted at the workplace or while they are working.” Negative effects on healthcare workers from WPV include immediate physical harm (Mento et al., 2020), low job satisfaction, cynicism and burnout as well as long-term physical symptoms such as depression, gastric problems, headaches, and muscles pains (Cánovas Pallarés et al., 2023). These effects lead to absenteeism, decreased productivity, increased employee health costs, and increased staffing turnover (Cabilan et al., 2022). Despite the health impact of WPV, there are limited evidence-based or publicized solutions available (Anderson et al., 2010).

Multiple risk factors have been identified that lead to violence in hospitals which include poor environment design, inadequate security and overcrowded workplaces (Lin et al., 2014). Within healthcare services, emergency departments (EDs) have up to four times higher risk of WPV than in other healthcare settings (Cánovas Pallarés et al., 2023; Pagnucci et al., 2022). The ED environment is prone to increased levels of aggression and violence in part due to the uncontrolled movement of the public, long wait times, overcrowded departments, patient and visitor stress levels and increasing numbers of patients presenting with substance abuse and mental health issues (Aljohani et al., 2021; Timmins & Timmins, 2021).

There is an increasing body of evidence suggesting that preventative and mitigating strategies that encompass the staff, patients and environment within EDs are needed to address WPV (Recsky et al., 2023; Renker et al., 2015). When considering ways to mitigate risk or increase safety, the “Hierarchy of Risk Controls” can be used to prioritize actions (Morris & Cannady, 2019). In the “Hierarchy of Risk Controls,” strategies that eliminate risk entirely by removing a potential hazard are given highest priority followed by substitution where a risky task or product is swapped out. Engineering controls such as physical barriers that limit the impact on staff and administrative controls such as staff education, or personal protective clothing are ranked as the lowest priority in the hierarchy of control measures (Morris & Cannady, 2019). In situations where risks cannot be eliminated, consideration should be given to options to reduce or manage risk before focusing mostly on training professionals to work around the risk (Morris & Cannady, 2019). To date, interventions used to reduce WPV have focused mostly on incident management and staff training (which are lower priorities on the hierarchy of risk) rather than prevention of violence (Renker et al., 2015; Spelten et al., 2020).

*When considering ways to mitigate risk or increase safety, the ‘Hierarchy of Risk Controls’ can be used to prioritise actions *

Within healthcare settings, physical design interventions have been shown to have a positive impact on work processes, especially in relation to safety and efficiency (Pati et al., 2012). Poor environmental design, including the space and equipment in which healthcare is delivered, has been identified as a risk factor impacting the incidence and severity of violence towards healthcare workers (MohammadiGorji et al., 2021). Physical and environmental designs are not clearly and consistently defined in the literature. For the purposes of this article we defined physical design as the built environment, including the layout, structure, esthetic, placement of furniture, and built-in physical elements within a particular space. Ensuring appropriate physical design of working environments should form an important part of WPV mitigation strategies (Aljohani et al., 2021) and assist employers meet their responsibility to provide a safe workplace environment for staff and patients (Beattie et al., 2020).

*Within healthcare settings, physical design interventions have been shown to have a positive impact on work processes*

A systematic review by Wirth et al. (2021) examined the research available on the evidence supporting implementation of interventions to reduce WPV. The review looked at a variety of diverse factors that influence violence including de-escalation training, self-defense classes and use of metal detectors. Wirth et al. (2021) found that most studies are unclear regarding the effectiveness of interventions and their impact on WPV. Most interventions were focused on the behavioral aspect of managing WPV, which is noted to be subordinate to environmental controls within risk management hierarchies. In a previous systematic review by Anderson et al. (2010), it was also identified that there are limited research studies to support that current interventions are effective in decreasing WPV. As this study is older than 15 years, there is a strong case for an updated understanding of the literature available. Spelten et al. (2020) recommend a move to organizational level supports and systems that do not focus on individual workers. Physical designs of spaces are a good example of interventions that do not require individual actors to undertake behaviors to impact on potential WPV incidents.

Changing a buildings design can be expensive, so effective design during the planning phase of health care facilities is essential in supporting the safety of patients and staff (Pati et al., 2016). It is essential that in designing health facilities, evidence-based design strategies for mitigating WPV are considered. Without clear evidence about the effectiveness of physical design to decrease violence, these decisions are being made on gut feelings or based on what have worked in the past (Reismann et al., 2023). The aim of this article is to examine the evidence for the effectiveness of physical design interventions to reduce WPV in EDs.

## Methods

A scoping review was selected to investigate whether there is evidence for the effectiveness of physical design interventions to reduce aggression in EDs. A scoping review was selected as these are used to identify and summarize available research in a particular area of study, to clarify key concepts and identify gaps in the literature (Akhter et al., 2023). The scoping review followed the process outlined by the Joanna Briggs Institute (JBI) methodology for scoping reviews (Peters et al., 2020). The scoping review process does not include a critical appraisal of studies evidentiary quality. Reporting was undertaken using the “PRISMA Extension for Scoping Reviews” checklist (Tricco et al., 2018).

### Inclusion and Exclusion Criteria

All available research evidence on the effect of physical design on violence in EDs was considered for this study. There were no restrictions placed on discipline of participants or publication dates, and only full text articles published in English were included. Papers were excluded if they related to mental health, pediatric EDs, discussion papers, non-research papers or literature reviews. Grey literature (organizational documentations not published through commercial processes) or unpublished papers were not included as part of the search strategy.

### Data Sources and Search Strategy

Following consultation with a university research librarian, a search was undertaken of the academic databases CINAHL, Medline, Scopus, and PsycINFO. The search utilized Boolean operators with key words, major search terms, and subject headings including aggression, violence, design, and ED. Studies were limited to intervention studies (see Table 1).

**Table 1.** Search Terms Related to Impact of Physical Design on Violence in Emergency Departments.

Search terms used
Intervention AND Aggres* OR “Aggress* behav*” OR violen* OR disturb* “disturb* behav*” OR “verbal aggress*” OR “physical aggress*” OR “confront* behav*” OR harrass* OR abus* OR “abus* behav*” OR “Workplace violence” OR “occupational violence” OR hostil* OR aggression OR violence AND ED* OR “emergency department” OR ER* OR casualty OR “Accident & emergency” OR “A & E” OR “Emergency service” AND Design* OR build* OR “built environment*” OR environment OR facilit* OR “design intervention*” OR “physical design” OR architecture OR MW “Facility design and construction” OR “Hospital design and construction” OR “Interior design and furnishings”

**Figure 1.**
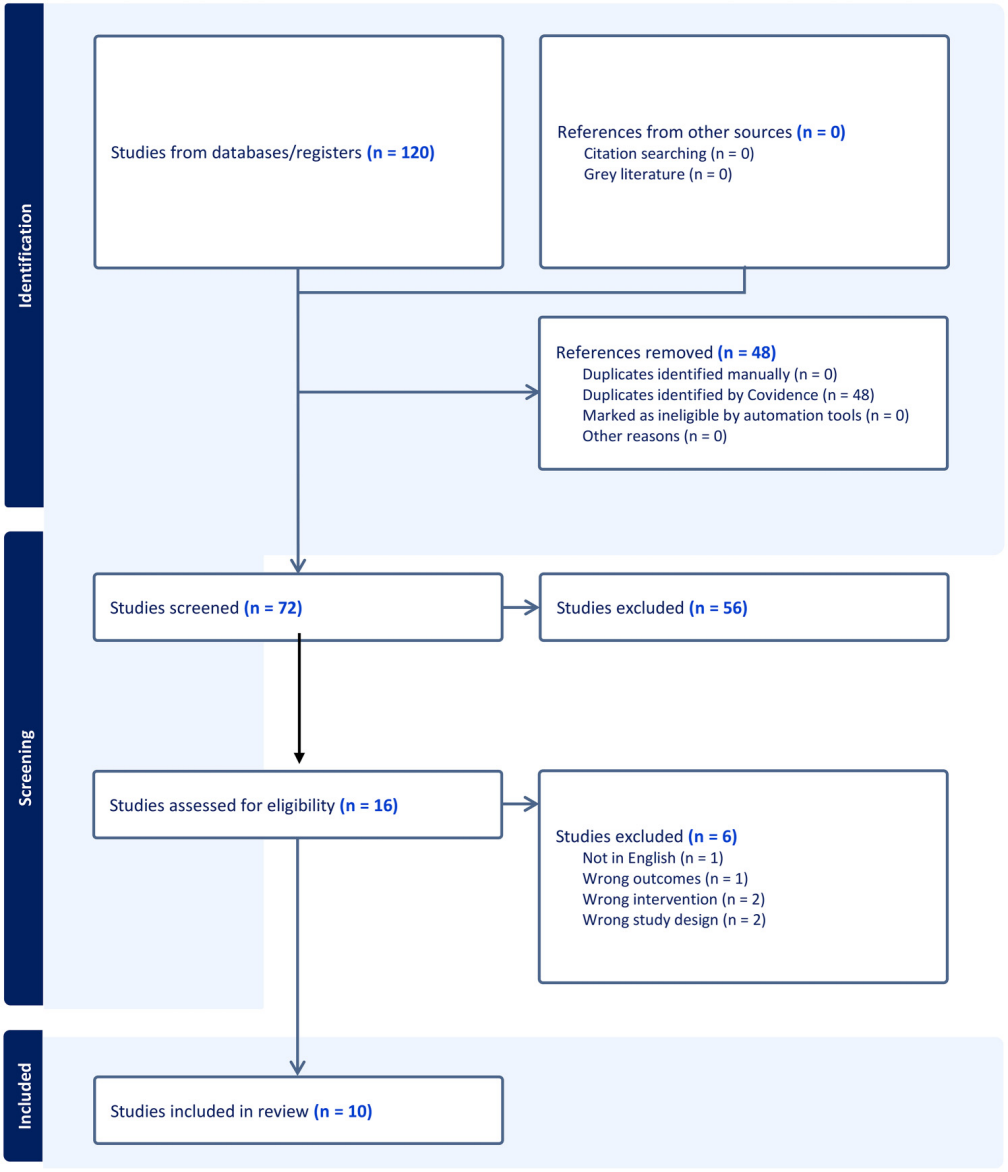
PRISMA Table.

A total of 21,345 overall results were obtained from the initial search. All results were then imported to Endnote. Due to the large number of results an automated AI screening program, ASreview (van de Schoot et al., 2021), was used to sort and filter the potential papers. This program sorts papers by relevance and then continuously re-sorts the papers order based on those accepted for review by the researchers (Quan et al., 2024). A heuristic stopping approach criteria was used, which determines the number of papers that are irrelevant before screening can be stopped (Callaghan & Müller-Hansen, 2020). The authors continued screening until they were able to go through 100 consecutive papers without identifying any additional relevant studies. Following this process the authors did not conduct further AI queries of the wider dataset. A total of 1002 results were reviewed in ASreview at abstract level by a single reviewer for relevance, using a generous attitude to inclusion. One hundred and twenty papers were identified relevant and imported into Covidence (Veritas Health Innovation, 2023) for review, of which 48 were duplicates. Covidence is an online program designed for undertaking systematic reviews (Veritas Health Innovation, 2023). The program enables multiple reviewers to screen title, abstract, and full text of articles and has data extraction and quality review sections to assist with the review. There were 72 results that were abstract screened by two reviewers with a resulting 16 articles screened at full text (see Figure 1). Any conflicts were resolved by a third reviewer.

### Data Extraction

Data extraction was undertaken using Covidence to identify content related to the effectiveness of physical interventions to reduce WPV. Data extracted included year, country, purpose, study population, study design and key findings related to physical design interventions (Table 2). Three reviewers independently extracted data from the studies and discussed the results to establish concordance. Any conflicts regarding data extraction were resolved by a fourth reviewer. Quality appraisal of the papers was not undertaken as the purpose of a scoping review is to catalogue the evidence that exists in a variety of formats rather than to provide a detailed analysis of the evidence quality (Peters et al., 2020).

**Table 2.** Summary of Evidence.

Author (year) Country	Aim of study	Study type	Participants	Physical design elements	Outcomes
Cabilan et al. (2022) Australia	To explore and collate solutions for occupational violence from emergency department (ED) staff	Cross-sectional study—electronic survey	*N* = 81	Lighting; Cameras; Placement of security/guards; Locks/Access restrictions; Location of entrance; Waiting room; Other: chalkboard walls, the orientation of the room, providing reading materials, puzzles, etc., use of duress alarms, managing overcrowding, absence of clutter specific cubicles for violent/aggressive patients- separate from other patients, removal of clutter or items that can be used as weapons, set egress routes, security/body cameras, weapons screening.	Prevention strategies included provision of comfort measures for patients, security presence, availability of duress alarms, removal of items that can be used as weapons, egress routes, security cameras, weapons screening, management of overcrowding, de-escalation rooms and controlled entry to ED.
d'Ettorre et al. (2020) Italy	To develop methodological technique for a preliminary assessment of type II workplace violence risk in EDs	Delphi method—questionnaire	*n* = 10: 5 occupational physicians, 2 clinical psychologists, 2 occupational therapists, 1 forensic physician.	Lighting; Cameras; Barriers; Nurses station; Waiting room; Room clutter; Other: security alarms, comfort and microclimate	18 organizational and environmental factors were found to be associated with WPV including the following aspects of physical design: placement of security officers, bright/effective lighting, spacious waiting room, closed circuit video, placement of nurses’ station, enclosed reception desk with bullet proof glass, de-escalation spaces, security alarm systems
El-Hadedy and El-Husseiny (2022) Egypt	To identify the environmental features that potentially influence WPV in the ED.	Case study using field observation combined with space syntax analyses of the spatial attributes and interviews	1 ED	Lighting; Cameras; Placement of security/guards; Barriers; Locks/Access restrictions; Location of entrance; Nurses station; Waiting room; Room clutter; Access to weapons; Room orientation; Entrances/exits; Other: patient areas are observable, but private, no blinds/hidden places in department, mirrors to monitor blind spots, panic alarms accessible, zones in waiting room for violent patients, furniture secured to floor/walls, metal detectors, segregated staff room, easy access to toilets, food/drink, phones	Crime prevention through environmental design may be applicable to the healthcare setting. Patient movement and visibility throughout EDs may impact WPV
Gates et al. (2011) USA	To plan, implement and evaluate a violence prevention and management intervention	Qualitative study- focus groups	Management (*n* = 24), Health service Employees (*n* = 47), patients (*n* = 25)	Placement of security/guards; Locks/Access restrictions; Waiting room; Entrances/exits; Other: isolation spaces, alert buttons, quiet areas, metal detectors	Violence may be prevented by improving patient comfort in ED waiting rooms and limiting access of bystanders. Perceptions of the effectiveness of metal detectors were conflicting. A lack of secure rooms was identified as issue in managing violent patients. The ability to isolate patients and access to panic buttons are important for managing violence.
Lin et al. (2014) Taiwan	To investigates the need for workplace improvement and provide potential solutions to minimize the risk of violence against HCWs in Taiwanese EDs.	Cross-sectional study using focus groups and interviews	Six sites with 6–10 health care workers at each	Cameras; Placement of security/guards; Barriers; Waiting room; Room clutter; Entrances/exits; Other: isolation rooms, panic alarms overcrowding, lack of boundaries waiting area, overcrowding	Workplace violence concerns can probably be attributed to design in the ED. Inappropriate design was found to provoke violence. Specific features of concern included: Lack of boundaries in waiting rooms and lack of emergency exits Strategies suggested to prevent violence: Control overcrowding in waiting rooms, design for family and relatives in waiting room, provide separation of patients to maintain privacy during waiting, separate rooms for mental health and substance abuse patients
Mahalleh and Fallahi (2019) Iran	To explore the effect of a workplace violence management program on the incidence of workplace violence against nurses at hospital EDs in Tehran	Quasi experimental and post intervention questionnaires	48 nurses intervention *n* = 24 control *n* = 24	Lighting; Cameras; Placement of security/guards; Ventilation	The frequency of psychological and physical violence was respectively 62.5% and 41.7% in the intervention group and 75% and 25% in the control group.
Pati et al. (2016) USA	To identify physical design attributes that potentially influence safety and efficiency of ED operations.	Qualitative research, gaming and interviews	Clients and contacts of a large architecture firm specializing in healthcare design. *N* = not stated	Placement of security/guards; Locks/Access restrictions; Location of entrance; Nurses station; Waiting room; Room clutter; Room orientation; Entrances/exits; Other: metal detectors, bullet proof glass, traffic management, centralization versus decentralization, special population areas.	An association exists between physical design attributes and ED security Five physical design attributes emerged substantially associated with security issues: the entry zone, traffic management, patient room clustering, centralization versus decentralization, and provisions for special populations.
Reismann et al. (2023) Germany	To explore how employees perceive measures to prevent violence and aggression in German EDs, with a focus on environmental, organizational, and individual-focused measures.	Qualitative interviews	The participants (*N* = 27) worked in 19 different Eds, doctors (*n* = 13) and nurses (*n* = 14).	Lighting; Cameras; Barriers; Locks/Access restrictions; Nurses station; Waiting room; Room clutter; Access to weapons; Entrances/exits; Other: alarms, calming wall colors, single rooms for violent/aggressive patients. The importance of day light in treatment rooms, seats were fixed to the walls, employee's workstations were kept on carts outside the room, short distances within the ED to prevent isolated work in secluded areas of the ED	Alarm systems (pagers and telephones), locking systems, camera surveillance perceived as effective in deterring violence and improving staff perception. Open plan design, calming wall colors, fixed furniture and equipment, glass encasement of workstations, and adequate escape doors can facilitate violence prevention. Old building are less effective at violence prevention due to constraints in altering spaces
Renker et al. (2015) USA	To identify and describe staff experiences, concerns and perceptions related to violence and abuse perpetrated by patients, family and non-family visitors in a level 1 ED.	Mixed methods using cross-sectional survey with interviews	Registered nurse = 41 Paramedics = 10 Not stated = 1 (84%, *n* = 41) were female	Cameras; Locks/Access restrictions; crowding; seclusion rooms; patient privacy; panic buttons; metal detectors; security signs; mirrors	Environmental changes could help minimize violence in the ED
Wong et al. (2017) USA	To describe the lived experience of staff members caring for this population to provides a broad perspective of ED patient violence	Qualitative interviews	*n* = 31 ED direct care staff, Hospital police (*n* = 9), Nurses (*n* = 10), Patient care technicians (*n* = 6), Emergency resident physicians (*n* = 6)	Lack of privacy; overcrowding	Environmental challenges and systems issues both in and outside the ED exacerbate threats to safety

### Data Analysis

The data analysis involved a three-step approach which included extraction, developing categories for findings and then synthesizing of findings to guide the review (Lockwood et al., 2015). This involved examining findings and identifying themes inductively. The team analyzed potential categories and themes drawn from the studies which required a thorough review of what physical design element was measured and the explicit rationale for the intervention. This process required multiple article readings and discussions to identify common themes from the articles.

## Results

Ten papers were identified that related specifically to interventions to manage WPV in EDs (see Table 2). Studies were from Australia, Egypt, Italy, Taiwan, Iran, Germany, and the United States of America. The studies used a variety of diverse methods including case study, cross-sectional surveys, mixed methods, quasi experimental and qualitative studies. All of the studies related to perceptions of staff of physical design on WPV. One study also measured the incidence of aggression rates following the implementation of physical design interventions such as ventilation, CCT TV, lighting modification to manage violence, although they reported no statistically significant outcome due to a low sample size and short time frame (Mahalleh & Fallahi, 2019). One case study identified where WPV occurred related to physical design features within four health services through observations (El-Hadedy & El-Husseiny, 2022). While it is acknowledged that only one paper discussed the implementation of physical design to decrease WPV, this paper has examined interventions that are seen to be effective for decreasing WPV in ED's.

The data revealed four main themes where physical design interventions could impact on violence and aggression from patients. These were: preventing harm from weapons, controlling physical access, observation and awareness, and patient comfort.

### Preventing Harm From the Use of Weapons

Eight papers identified preventing harm from weapons as an important factor in managing violence and aggressions (Cabilan et al., 2022; d'Ettorre et al., 2020; El-Hadedy & El-Husseiny, 2022; Gates et al., 2011; Mahalleh & Fallahi, 2019; Pati et al., 2016; Reismann et al., 2023; Renker et al., 2015). Weapon screening can be enabled by appropriately designed physical spaces (Cabilan et al., 2022; El-Hadedy & El-Husseiny, 2022; Gates et al., 2011; Pati et al., 2016; Renker et al., 2015), securing items that can be used as weapons (Cabilan et al., 2022; El-Hadedy & El-Husseiny, 2022; Mahalleh & Fallahi, 2019; Reismann et al., 2023) and stopping weapons from being able to be used against staff, for example by the use of bullet proof screens (d'Ettorre et al., 2020; El-Hadedy & El-Husseiny, 2022; Pati et al., 2016; Renker et al., 2015). Differences in opinion on the effectiveness of screening for weapons was noted by several studies (Gates et al., 2011; Renker et al., 2015). One paper reported that participants felt the use of metal detectors may increase weapons related incidents by lulling people into a false sense of security and others suggesting they only be used during heightened security events (Pati et al., 2016).

### Controlling Physical Access

Controlling physical access can include limiting access to areas for specific people (El-Hadedy & El-Husseiny, 2022; Pati et al., 2016; Reismann et al., 2023; Renker et al., 2015). This was also termed “natural access control” (El-Hadedy & El-Husseiny, 2022). Controlling the entry and exits was viewed as a physical design feature that could help decrease violence from both patients and family members (Mahalleh & Fallahi, 2019). Control of physical access to the ED and its different areas can involve using electronic or manual locks on doors, (El-Hadedy & El-Husseiny, 2022; Pati et al., 2016; Reismann et al., 2023; Renker et al., 2015), keypads, proximity cards, or biometric readers (Cabilan et al., 2022; El-Hadedy & El-Husseiny, 2022; Gates et al., 2011; Lin et al., 2014; Pati et al., 2016; Reismann et al., 2023; Mahalleh & Fallahi, 2019). One study reported that coded locks in ED were not efficient (Renker et al., 2015). Exit doors and doors between areas were expected to open only from the inside to prevent unauthorized access (El-Hadedy & El-Husseiny, 2022; Pati et al., 2016). Physical barriers, such as using lines on floors to outline areas, counters, stations, gates (El-Hadedy and El-Husseiny (2022) can be used to define property lines and are part of the “Crime Prevention Through Environmental Design” strategy for physical management to deter crime in specific areas.

Studies discussed the importance of egress routes and having access to a means of quick exit such as specifically allocated routes or a lockable escape room to isolate from violent and aggressive incidents (Cabilan et al., 2022; Lin et al., 2014; Reismann et al., 2023). De-escalation and waiting rooms require clearly signed entry and exit routes to ensure they are visible (Cabilan et al., 2022; El-Hadedy & El-Husseiny, 2022). It was seen as preferable to have separate entrances to ED's for patients and relatives than those used by healthcare staff (El-Hadedy & El-Husseiny, 2022).

Patient flow is considered a violence prevention measure that is impacted by how the building is designed. The positioning of doors, walls, signage, furniture and exit points can all have an impact on patient flow (El-Hadedy & El-Husseiny, 2022; Pati et al., 2016; Reismann et al., 2023; Renker et al., 2015). Flow could be controlled by patients moving only forward (one direction) in their patient journey (Reismann et al., 2023). This may help to prevent patient dissatisfaction and potential aggressive incidents occurring (Pati et al., 2016). Having multiple waiting rooms to separate different acuity levels of patients (Pati et al., 2016) was preferred.

### Observation and Awareness

Observation and awareness refer to how physical design impacts the ability of staff to see and monitor patients and relatives and obtain assistance when violence occurs. Nine papers mentioned the importance of healthcare staff having visibility to monitor patients and relatives to ensure the ability for early intervention of violence and aggression (Cabilan et al., 2022; d'Ettorre et al., 2020; El-Hadedy & El-Husseiny, 2022; Gates et al., 2011; Lin et al., 2014; Pati et al., 2016; Reismann et al., 2023; Renker et al., 2015; Mahalleh & Fallahi, 2019). It is suggested the patients should be easily visualized by staff in all work areas including reception, waiting rooms and areas inside and immediately outside the ED (Cabilan et al., 2022; El-Hadedy & El-Husseiny, 2022). Uninterrupted line of sight with an open design increases staff awareness on what is happening around them (Pati et al., 2016). The ability to observe all patients in the waiting room and separate people if necessary are natural surveillance measures that make waiting rooms safer (El-Hadedy & El-Husseiny, 2022). Pati et al. (2016) suggested that open waiting rooms provide better visual and auditory access, with a caveat that for some special care populations, rooms with restricted visibility provide a low stimulus environment and may assist with behavioral management. Visibility is also influenced by the design of the department. For example, having small or large pod arrangements, glass panels were fitted in doors and walls to allow monitoring of potential blind spots (El-Hadedy & El-Husseiny, 2022). Smaller pod designs make help seeking more difficult due to isolation and decreases visibility compared to a larger pod layout (Pati et al., 2016; Renker et al., 2015). Patient rooms with limited or restricted line of sight to the nurse's station or restricted visibility between work areas, can impact the security of patients and staff (Pati et al., 2016).

Surveillance was seen as important part of managing aggression and violence in eight of the studies (Cabilan et al., 2022; d'Ettorre et al., 2020; El-Hadedy & El-Husseiny, 2022; Lin et al., 2014; Pati et al., 2016; Reismann et al., 2023; Renker et al., 2015; Mahalleh & Fallahi, 2019). Natural surveillance uses the environment to give the best opportunity for observing an area and includes design features such as placement of openings, windows, buildings and entrance orientation to enhance safety of the environment and those that use it (El-Hadedy & El-Husseiny, 2022). Using camera monitoring was an important part of surveillance measures inside and outside of the ED and was perceived as a deterrent to reduce WPV occurrences (Cabilan et al., 2022; D’Ettore et al., 2020; El-Hadedy & El-Husseiny, 2022; Lin et al., 2014; Mahalleh & Fallahi, 2019; Pati et al., 2016; Reismann et al., 2023; Renker et al., 2015). Surveillance equipment must be operable and in the right locations (Lin et al., 2014). One study suggested that camera surveillance could be seen as a deterrent only if signposted (Reismann et al., 2023). Camera monitoring could be retrofitted if it had not been included in the original building design (Pati et al., 2016).

Lighting was seen as an important factor to decrease WPV (Cabilan et al., 2022; d'Ettorre et al., 2020; El-Hadedy & El-Husseiny, 2022; Pati et al., 2016; Reismann et al., 2023; Mahalleh & Fallahi, 2019), although studies disagreed as to whether increasing lighting to improve surveillance (D'Ettorre et al., 2018; El-Hadedy & El-Husseiny, 2022; Mahalleh & Fallahi, 2019) or decreasing light to improve patient comfort had the best impact on WPV (Cabilan et al., 2022). Day light in treatment rooms was also considered important to prevent patients becoming disorientated, and hence help prevent WPV (Reismann et al., 2023).

Location of security staff bases were seen to impact on WPV (Cabilan et al., 2022; El-Hadedy & El-Husseiny, 2022; Pati et al., 2016; Reismann et al., 2023). It was suggested that security should be located at the main hospital entrance to help with unauthorized access and provide staff reassurance (El-Hadedy & El-Husseiny, 2022; Reismann et al., 2023) or near the main ED (Cabilan et al., 2022; Lin et al., 2014). Locating security within the ED lobby close to the nurse's station enables the security staff to have a direct line of site to the entry area and was seen to provide an increased security presence to decrease WPV (Lin et al., 2014).

Duress alarms, panic button access and security alarms were seen as important strategies to manage WPV when incidents were imminent or occurring (Cabilan et al., 2022; El-Hadedy & El-Husseiny, 2022; Gates et al., 2011; Lin et al., 2014; Reismann et al., 2023; Renker et al., 2015). This included the positioning of duress alarms close to or within patient rooms (Cabilan et al., 2022) and was seen to be part of natural surveillance measures (El-Hadedy & El-Husseiny, 2022). Some health services reported having duress alarms that are connected to police stations (Lin et al., 2014). According to Renker et al. (2015) participants ranked panic button/silent alarm as being very effective and endorsed this as helping with staff safety.

### Patient Comfort

The final category “patient comfort” related to the impact of design elements that prioritized patient comfort and the effect they can have on WPV (Cabilan et al., 2022; d'Ettorre et al., 2020; El-Hadedy & El-Husseiny, 2022; Gates et al., 2011; Lin et al., 2014; Pati et al., 2016; Reismann et al., 2023; Renker et al., 2015; Wong et al., 2017).

Quiet spaces, also called de-escalation or seclusion rooms, that allow patients privacy and reduce noise were frequently referred to as a preventative measure (Cabilan et al., 2022; d'Ettorre et al., 2020; El-Hadedy & El-Husseiny, 2022; Gates et al., 2011; Lin et al., 2014; Reismann et al., 2023; Renker et al., 2015) and can also be used as a safe room for aggressive patients (Cabilan et al., 2022; Gates et al., 2011). These specially built single rooms or specialized rooms with lockable doors can be used to provide individualized care to patients who require a low stimulus calming environment and can help to care for those with behavioral problems such as psychiatric and substance abuse issues (Cabilan et al., 2022). Although most EDs have these areas, some do not have an appropriate number to cater for theses patient cohorts (Gates et al., 2011; Reismann et al., 2023; Renker et al., 2015). There are strong staff perceptions that these rooms provide protection for staff and other patients from challenging behavior (Reismann et al., 2023).

Waiting room design was seen to impact on WPV (Cabilan et al., 2022; d'Ettorre et al., 2020; El-Hadedy & El-Husseiny, 2022; Gates et al., 2011; Lin et al., 2014; Pati et al., 2016; Reismann et al., 2023; Renker et al., 2015). Suggested violence prevention measures in waiting rooms include an open and welcoming design with soothing wall colors (Lin et al., 2014; Reismann et al., 2023). Cabilan et al. (2022) suggested that chalk board walls can be used for distraction therapy to help ease stress while waiting. Some escalation in behavior may be due to a lack of access to food and drinks (Cabilan et al., 2022; Reismann et al., 2023), hence waiting rooms should have easily accessible toilets, food, drink and public phone that are well signposted and properly maintained (El-Hadedy & El-Husseiny, 2022; Gates et al., 2011). Another suggestion by Reismann et al. (2023) was to design different waiting rooms so the patients move forward and never return to the same waiting room to alleviate frustration and potential aggression. If separate waiting areas are used it is essential that they have clear signage (El-Hadedy & El-Husseiny, 2022).

Overcrowding of the ED waiting room was mentioned as a cause for WPV in seven papers (Cabilan et al., 2022; d'Ettorre et al., 2020; Lin et al., 2014; Pati et al., 2016; Reismann et al., 2023; Renker et al., 2015; Wong et al., 2017). Overcrowding resulting in long waiting times was seen as a main risk factor for violence (Pati et al., 2016; Reismann et al., 2023; Renker et al., 2015). Overcrowding decreases privacy of patients in waiting rooms and may increase the difficulty of managing agitated patients (Wong et al., 2017). Ensuring that EDs have appropriate space in waiting areas may assist with managing WPV (Cabilan et al., 2022).

## Discussion

This scoping review has identified that there is limited evidence to show the effectiveness of any physical design elements to reduce WPV in hospital ED's. However, there is evidence that ED staff perceive physical design impacts on their safety. In hospital EDs, physical design of spaces has been connected to preventing harm from weapons. Physical design can also impact on the ability to control access to spaces, the ability to observe spaces and the comfort of patients within those spaces. The physical design interventions identified that may reduce WPV range from micro level designs such as cameras, lighting and access controls to macro level designs such as placement of walls and design of the workplace (open design or decentralized features) (Pati et al., 2016). Designing EDs with adequate space between patients can increase personal privacy and reduce potential conflicts (Lin et al., 2014).

*There is evidence that ED staff perceive physical design impacts on their safety.*

Physical spaces are not neutral. According to Pati et al. (2016) the way spaces are designed and setup are inherently linked to the processes and actions that are able to occur within that space, and the ease with which they can be conducted. EDs are by nature noisy and chaotic, as they are filled with people who are experiencing emergency situations (Timmins & Timmins, 2021). Maintaining patient privacy in a mostly open environment while treating aggressive patients can be difficult, with design elements such as the availability of seclusion rooms impacting on the ability to manage these situations appropriately (El-Hadedy & El-Husseiny, 2022).

*Physical spaces are not neutral. *

Any changes made to the design of physical spaces should be assessed to ensure there are no unintended consequences. For example, installing security cameras with visible signage may reduce WPV but have negative impacts on personal privacy of staff and patients receiving care (Reismann et al., 2023). Two studies included in this review also discussed the way that interventions to eliminate or reduce risk may provide a false sense of security (Renker et al., 2015; Gates et al., 2011).

### The Organizational Cost of Workplace Violence

Healthcare providers have a vested interest in finding interventions that can reduce the rates and severity of incidences of violence towards staff because they face significant organizational costs when violent incidents do occur (Chong et al., 2015). A systematic review by Hassard et al. (2019) identified many organizational costs from WPV including financial, injury, distress and psychological harm. The impacts for staff related to physical, emotional and personal stress may impact their ability to perform their work and can result in ongoing costs to organizations from decreased job performance, satisfaction, retention and staff moral (Speroni et al., 2014). Research into ways to manage patient aggression is essential, since there is a high cost of not managing WPV.

It is well established that WPV occurs in healthcare settings, but the evidence to support interventions to manage or reduce WPV is less well developed (Renker et al., 2015). This lack of evidence may cause difficulties for hospital administrators and decision makers who are seeking to ensure that WPV prevention is considered in renovations or building of new facilities. Reismann et al. (2023) has found that hospital managers may implement interventions that are not based on evidence because the evidence does not exist and they have a need to act rather than wait for evidence to be established so as meet the requirements for maintaining staff safety. An increased focus on providing evidenced based research to support the design of the environmental to assist in managing WPV is recommended (Renker et al., 2015; Spelten et al., 2020).

## Limitations

The authors acknowledge that this review may not have captured all interdisciplinary perspectives as the search strategy was focused on EDs which may have precluded some interdisciplinary research. All studies considered staff perception. While staff perceptions are valuable, self-reporting has limitations and may present biases that need to be considered such as selection bias, recall errors and the data may limit generalization (Brutus et al., 2012). Selection bias in perception studies may have occurred with staff who have a particular experience of WPV responding to surveys, which may not represent the view of the wider cohort. Self-reporting of the data may have introduced biases in the studies as experiences of WPV aggression may have been downplayed or exaggerated and cannot be independently verified.

## Recommendations

With limited research outlining the effectiveness of design-based interventions it is difficult to design ED spaces to manage aggression. The authors recommend further research be conducted around the impact of physical design elements on WPV, including pre-post studies, with established measures of effectiveness. Collecting and utilizing empirical evidence through incident data audits could reduce the limitations associated with individual perceptions and allow myths around space to be challenged (Thomas et al., 2024). To further enhance understanding of how physical design impacts on patient aggression, studies could include the use of systems integration simulations to identify how different environmental modifications impact workflow, staff movement, and patient interactions in real-time scenarios. Human factors design collaborations should also be considered when researching the effect of physical design on WPV in order to evaluate the interaction between environmental features and human behavior. It would be important for any suggested design intervention to evaluate cost-effectiveness to support evidence-based decision making for health service administrators. This could be done through the assessment of changes in staff Workcover compensation or other systems-based measures of patient violence impact.

## Conclusion

There is a legislated requirement to provide a safe workplace but minimal evidence to support the effectiveness of physical design to reduce aggression in ED's. Decisions are being made to redesign EDs based on opinion rather than evidence, in an effort to be seen to be doing something about the increasing problem of WPV. As this paper was only able to identify a small number of studies, and the evidence provided was not able to establish objective measures of effectiveness, no conclusions about the actual impact of any specific physical design elements on WPV can be provided. Research is required to provide evidence-based interventions to enable healthcare administrators to make informed decisions to guide policy and practice decisions to aid in preventing WPV in EDs.

## Implications for Practice

- violence and aggression in emergency departments is an increasing problem which may be mitigated by the physical design of the department
- physical design may impact on violence and aggression by preventing harm from weapons, controlling physical access, improving observation and awareness and aiding patient comfort
- further research is needed to investigate the effect of physical design on violence in emergency departments.

## Supplemental Material

sj-docx-1-her-10.1177_19375867251351027 - Supplemental material for Effectiveness of Environmental Design Interventions to Reduce Aggression and Violence in Emergency Departments: A Scoping Review Supplemental material, sj-docx-1-her-10.1177_19375867251351027 for Effectiveness of Environmental Design Interventions to Reduce Aggression and Violence in Emergency Departments: A Scoping Review by Darren Jacob, Belinda Jacob, Elisabeth Jacob and Alycia Jacob in HERD: Health Environments Research & Design Journal
